# Unexplained colonic necrosis in a patient with end-stage kidney disease on chronic hemodialysis: case report and review of uremic colitis

**DOI:** 10.1186/s12871-024-02639-x

**Published:** 2024-07-23

**Authors:** Jing Zhou, Yisen Zeng, Xiaoying Zhou, Yong Liu

**Affiliations:** Department of Critical Care Medicine, Zhangzhou Affiliated Hospital of Fujian Medical University, Zhangzhou, 36300 Fujian China

**Keywords:** Uraemia, Haemodialysis, Intestinal necrosis

## Abstract

**Background:**

Intestinal necrosis in uremic patients has been reported but is rare.

**Case presentation:**

A 56-year-old male patient who underwent long-term regular haemodialysis was admitted to the hospital due to involuntary shaking of the limbs and nonsense speech. The patient’s symptoms improved after continuous blood purification under heparin anticoagulation, rehydration, sedation, and correction of electrolyte disturbances. However, the patient experienced a sudden onset of abdominal pain and a rapid decrease in blood pressure; high-dose norepinephrine were required to maintain his blood pressure. A plain abdominal radiograph performed at bedside showed intestinal dilation. Colonoscopy revealed inflammation and oedema of the entire colon, with purulent secretions and multiple areas of patchy necrosis. The cause of intestinal ischaemia was not clear.

**Conclusions:**

Although rare, previous causes of uremic colitis have been reported. As the patient developed abdominal pain before the onset of shock and the necrosis was seen on colonoscopy, we suspect that this is a case of fulminant uremic colitis.

## Background

Chronic kidney disease (CKD) is a major global public health problem, affecting approximately 350 million people worldwide and resulting in 500,000 to 1 million deaths every year [1]. People with CKD require more medical and social care. Previous studies have suggested that patients with uraemia have common complications, such as cardiovascular disease, infection and cerebrovascular accidents [2–4], but there are few studies on intestinal problems in these patients. Many uraemic toxins may induce intestinal inflammation, which leads to intestinal motility disorders. However, little is known about the relationship between uraemia and intestinal inflammation [5]. Previous studies have suggested that the common causes of intestinal necrosis are low cardiac output, atherosclerosis, thrombosis, and drug use [6]. A model for the cause of superior mesenteric artery syndrome has also been reported [7]. Unexplained intestinal necrosis in patients with uraemia has not been reported. This article reports the case of a patient on maintenance haemodialysis who had total colonic lesions characterized by scattered flaky necrosis resulting in refractory shock. Additionally, this article reviews the literature on intestinal necrosis in uraemia patients.

## Case presentation

**Fig. 1:**
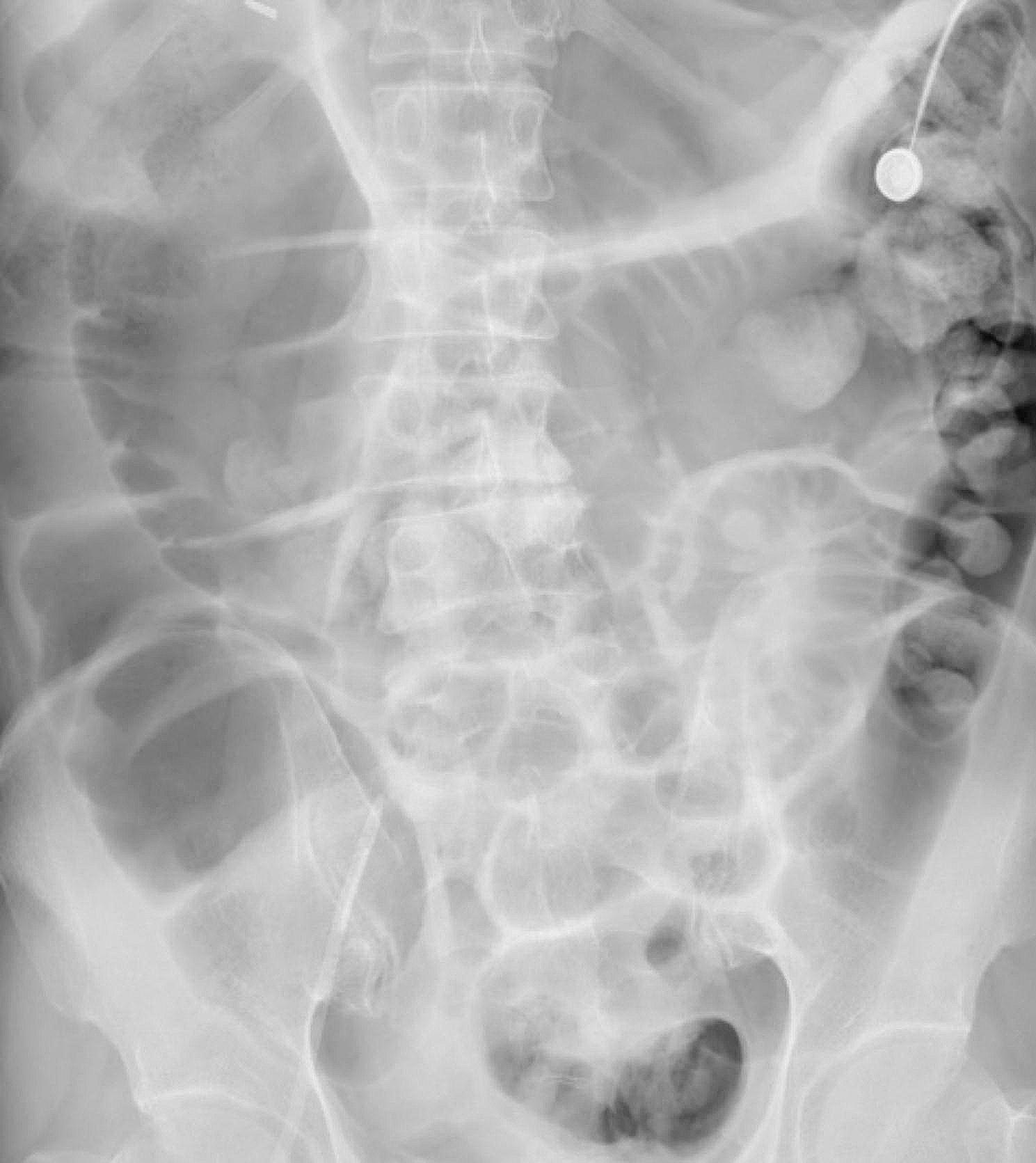
Plain abdominal radiography showing a distribution of scattered intestinal gas and contents, partial intestinal dilation

**Fig. 2:**
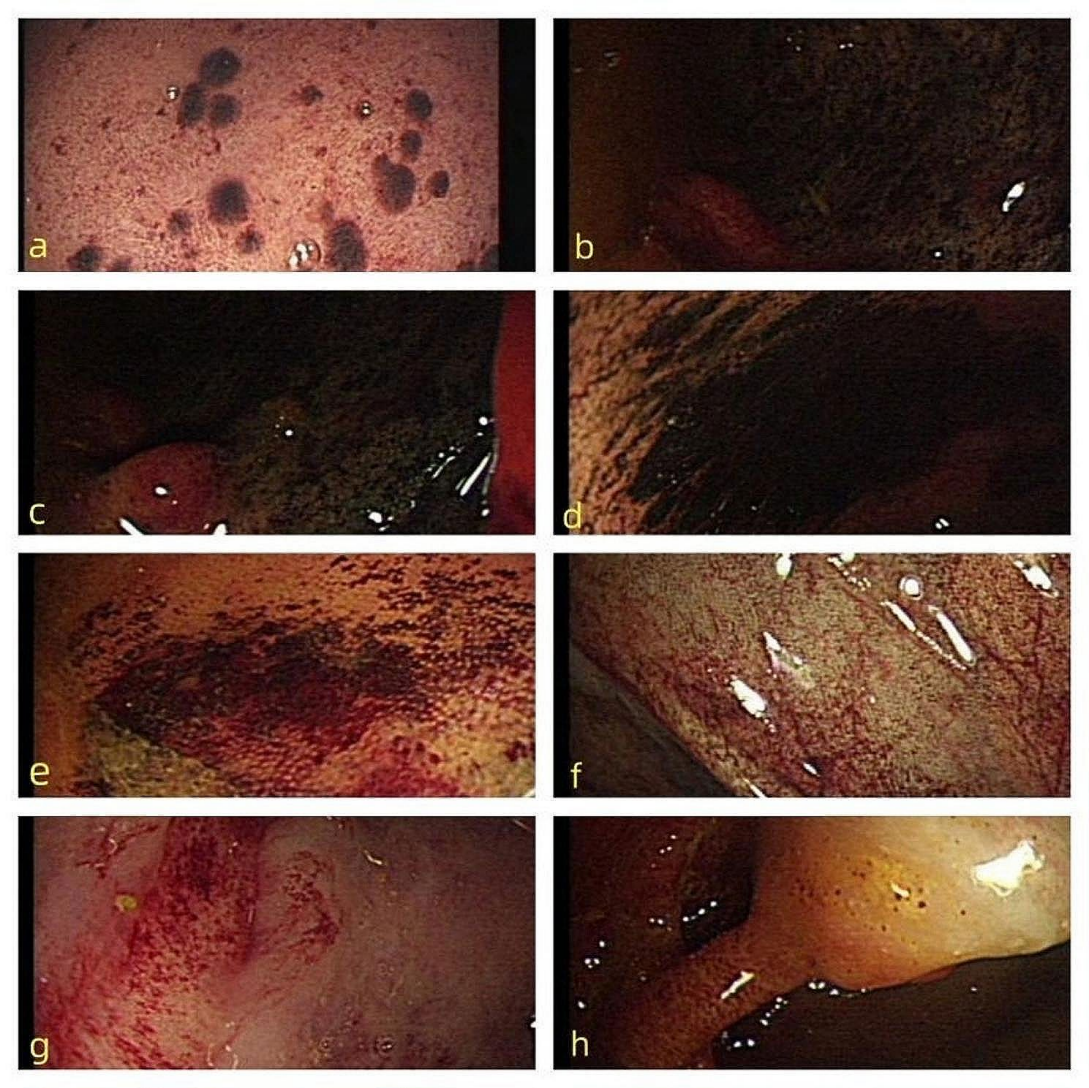
Colonoscopy revealed diffuse congestion and oedema of the whole colon mucosa and an abundance of purulent secretions. The colonic mucosa had scattered red, purple, and black patches, especially in the ileocecal mucosa, and long pedicle polyps were observed in the sigmoid. **a**, Rectum; **b**, ileocecal junction; **c**, ascending colon; **d**, transverse colon; **e**, descending colon; **f**, splenic flexure of colon; **g**, sigmoid colon; **h**, sigmoid colon

The patient, a 56-year-old male, was diagnosed with chronic glomerulonephritis and chronic kidney disease 9 years previously. He began maintenance haemodialysis 4 years prior and underwent haemodialysis 3 times a week. He had constipation, but he did not have diabetes, connective tissue disease, or heart disease. One day before hospitalization, he developed involuntary shaking of the limbs, dizziness, and incoherent writing and talking without obvious induction. The results of the tests performed on admission included the following: total white blood cells, 6.02 × 10^9/L (reference range, 4–10 × 10^9/L); neutrophils, 78.2% (reference range, 50-75%); haemoglobin, 108 g/L (reference range, 120–160 g/L); total platelets, 172 × 10^9/L (reference range, 100–300 × 10^9/L); potassium, 5.40 mmol/L (reference range, 3.5–5.5 mmol/L); sodium, 152.6 mmol/L (reference range, 135–145 mmol/L); calcium, 3.08 mmol/L (reference range, 2.1–2.7 mmol/L); phosphorus, 1.69 mmol/L (reference range, 0.7–1.6 mmol/L); serum creatinine, 1080.20 µmol/L (reference range, 40–133 µmol/L); urea nitrogen,17.4 mmol/L (reference range, 2-6.9 mmol/L); procalcitonin, 1.27 ng/ml (reference value, < 0.05 ng/ml); parathyroid hormone, 384.5 pg/ml (reference range, 12–88 pg/ml); and D-dimer, 856.00 ng/ml (reference range, 0-256 ng/ml). The patient’s liver function, myocardial enzyme levels, and coagulation test results were normal. Electrocardiography and colour Doppler ultrasound of the liver, gallbladder, pancreas and spleen were normal. Chest computed tomography (CT) revealed striped shadows in both lower lungs. The treatments included nifedipine tablets, clonidine tablets, methylcobalamin dispersible tablets, and haemodialysis. On the second day of hospitalization, the patient became restless with violent tendencies and was transferred to the intensive care unit (ICU). Blood creatinine was 1205.40 µmol/L (reference range, 40–133 µmol/L), urea nitrogen was 13.7 mmol/L (reference range, 2-6.9 mmol/L), calcium was 2.71 mmol/L (reference range, 2.1–2.7 mmol/L), and phosphorus was 1.87 mmol/L (reference range, 0.7–1.6 mmol/L). The patient was treated with continuous blood purification under heparin anticoagulation, sedation with midazolam, and dextrose via injection. The patient was conscious and cooperative and had good limb movement. On the sixth day of hospitalization, the patient experienced sudden abdominal pain, followed by shock and confusion 6 h later, and his blood pressure was maintained using large doses of vasopressor drugs. Physical examination revealed no obvious abnormalities in the heart or lungs, slight tension in the abdominal muscles, and no tenderness or rebound pain; the patient did not cooperate with the examination. Bowel sounds were heard 3 times/min. Emergency myocardial enzymes, troponin, brain natriuretic peptide (BNP), procalcitonin, blood culture, heart colour ultrasound, bedside chest and abdominal plain film, chest and abdominal colour ultrasound, electrocardiogram, and liver and kidney function were reexamined. The patient had a total white blood cell count of 15.27 × 10^9/L, a neutrophil ratio of 80.00%, a haemoglobin concentration of 143 g/L, a total platelet count of 218 × 10^9/L, a myoglobin concentration > 1000.00 ng/ml (reference value, < 110 ng/ml), a hypersensitive troponin I concentration of 787.72 ng/L (reference value, < 46.47 ng/L), a procalcitonin concentration of 2.72 ng/ml, a plasma fibrin degradation product concentration of 39.80 mg/L (reference value, < 5 mg/L), a D-dimer concentration of 7934.00 ng/mL, a glutamic oxalacetic transaminase concentration of 2119.5 U/L (reference range, 1–42 U/L), and a glutamic pyruvic transaminase concentration of 2060.0 U/L (reference range, 5–42 U/L). His cardiac troponin and bilirubin levels were not abnormal. An electrocardiogram (ECG) showed a sinus rhythm, but the patient had QT interval prolongation. An echocardiogram revealed an ejection fraction (EF) of 56%, and his cardiac systolic and diastolic function were normal. Abdominal colour ultrasound revealed abdominal distension, weak intestinal peristalsis, and intestinal dilation. Plain abdominal radiography revealed distribution of scattered intestinal gas and contents, partial intestinal dilation, no demarcation of the liquid level, a clear abdominal fat line, and no free gas under both sides of the diaphragm (Fig. 1). Chest radiography revealed a few flocculent shadows and fewer cord shadows in both lower lung fields than before treatment. A diagnosis of septic shock was considered, and the source of infection was hypothesized to be an infection within the abdomen. He was treated with an artificial airway, mechanical ventilation, anti-infection agents, crystalloids, vasopressor agents, acid inhibition agents, gastro-intestinal decompression, somatostatin, sufentanil and midazolam. The patient’s consciousness improved, but he still experienced abdominal pain and tightness. Additionally, the patient’s blood pressure was unable to be sufficiently controlled to allow him to safely undergo an enhanced abdominal CT examination. Colonoscopy revealed a substantial amount of faecal water and faeces in the whole colon, diffuse congestion and oedema of the whole colon mucosa, and abundant purulent secretions. The colonic mucosa had red, purple, and black scattered patches, especially in the ileocecal mucosa, and the appendiceal opening was not observed due to the poor visual field. Long pedicle polyps were observed in the sigmoid but did not reach the back end (Fig. 2). The patient’s lactic acid level was progressively elevated; his blood pressure was difficult to maintain, resulting in eventual failure of circulation and death.

## Discussion and conclusions

Cases of intestinal necrosis in uraemia patients were first reported many years ago [8, 9]. Risk factors for ischaemic colitis in nonuraemic patients include low cardiac output due to intrinsic cardiac causes (heart failure, myocardial infarction, arrhythmia) or extrinsic factors (sepsis, hypovolemia, shock). Vascular risk factors include atherosclerosis, vasculitis, and microembolism. Ischaemic colitis in nonuraemic patients can be caused by several drugs, such as digoxin, vasopressors, diuretics, and oestrogens [6, 10–13]. Constipation and colonic distension have also been reported [14]. However, ischaemic colitis can also occur without any known trigger. All risk factors for colon necrosis are applicable to both uraemic patients and nonuraemic patients [15]. In addition, it has been reported that the combination of sorbitol and sodium polystyrene sulfonate is associated with intestinal necrosis in uraemia patients [16].

An enhanced abdominal CT scan can help pinpoint the cause of ischaemia. Colonoscopy is a very sensitive and specific way to assess colon ischaemia but does not always allow a doctor to make a definitive diagnosis; however, biopsy can distinguish between inflammatory, infectious, and ischaemic colitis. In early ischaemia, the mucosa may appear pale and oedematous with scattered congested areas. Severe ischaemia may be characterized by submucosal oedema, which is characterized by blue or black nodules, and bleeding. These submucosal lesions are most commonly observed in the first 2–3 days after an ischaemic attack and resolve rapidly. Colonoscopy should be performed as early as possible, preferably within the first 3 days after symptom onset, to ensure optimal diagnostic potential [6, 17–19].

In this case, the patient was transferred to the ICU due to delirium. After adequate haemodialysis, the patient’s neurological symptoms disappeared. Cerebral angiography performed two years earlier revealed no plaque or stenosis in the arteries, and uraemic encephalopathy was suspected. No abdominal distension was found during the physical examination, no painful expression was found when pressing the abdomen, and bowel sounds could be heard. Therefore, the abdomen was not the focus of treatment. During the 4 days of ICU treatment, the patient received two enemas to stimulate bowel movements, after which the patient passed stools. An abundance of intestinal gas was observed on plain abdominal film. The patient had total colonic oedema and patchy mucosal necrosis in the sigmoid colon, descending colon, transverse colon, and ascending colon to the end of the caecum. The colonoscopist speculated that the patient had the same problem in the entire digestive tract, and the surgeon was unable to operate. The patient did not develop hypotension during haemodialysis, and shock developed only after the onset of abdominal pain. The patient had history of constipation, had no history of diabetes, heart disease or connective tissue disease. After transfer to the ICU, continuous heparin anticoagulation was performed, and the D-dimer concentration was less than 1000 ng/mL. Unfortunately, no pathology was performed to determine the cause of his intestinal lesions. Previous studies have shown that ischaemic enteritis is characterized by segmental necrotic changes. This patient’s colonoscopy revealed a lesion that affected his entire colon, which has not been previously reported. The cause of this patient’s condition may not have been any of the above mentioned causes of ischaemic enteritis. Uremic colitis has been reported [20–22] and there is (very old) experimental evidence that uremia can cause colitis [23]. A previous study of endoscopy in patients with CKD suggested that ischaemic colitis may occur in up to 3% of patients with CKD [24], and an autopsy study of 78 patients with ESKD on haemodialysis suggested that colitis is common (although severe colitis is rare) [25]. We suspect that this is a case of fulminant uremic colitis.

The article has limitations.The lack of histopathology and the lack of advanced imaging (e.g. computed tomography) are major limitations of this case report. Using the visual appearance of the mucosa during colonoscopy to try to make a diagnosis introduces considerable uncertainty.The patient developed abdominal pain before the onset of shock and the necrosis seen on colonoscopy, but it cannot be ruled out that this was an atypical case of ischaemic colitis due to subclinical shock that became apparent only after the patient developed abdominal pain.Clostridioides (Clostridium) difficile colitis was not tested. This, of course, can cause life-threatening pancolitis and refractory shock.

## Data Availability

No datasets were generated or analysed during the current study.
